# Dimethyl 3,3′-diphenyl-2,2′-[(*S*)-thio­phene-2,5-diylbis(carbonyl­aza­nedi­yl)]dipropano­ate

**DOI:** 10.1107/S1600536810033210

**Published:** 2010-08-25

**Authors:** Guang-Ming Xia, Jing Liu, Zhen Li, Mu-Wei Ji, Guo-Xin Sun

**Affiliations:** a Shandong Provincial Key Laboratory of Fluorine Chemistry and Chemical Materials, School of Chemistry and Chemical Engineering, University of Jinan, Ji’nan 250022, People’s Republic of China

## Abstract

The asymmetric unit of the title compound, C_26_H_26_N_2_O_6_S, contains two independent mol­ecules; each has twofold symmetry with the S atom and the mid-point of the C—C bond of the thio­phene ring located on a twofold rotation axis. In the two mol­ecules, the terminal benzene rings are oriented at dihedral angles of 65.8 (3) and 63.5 (3)° with respect to the central thio­phene rings. The meth­oxy­carbonyl group of one mol­ecule is disordered over two positions with site-occupancy factors of 0.277 (12) and 0.723 (12). Inter­molecular N—H⋯O hydrogen bonding is present in the crystal structure.

## Related literature

For applications of thio­phene derivatives, see: Xia *et al.* (2010 ▶).
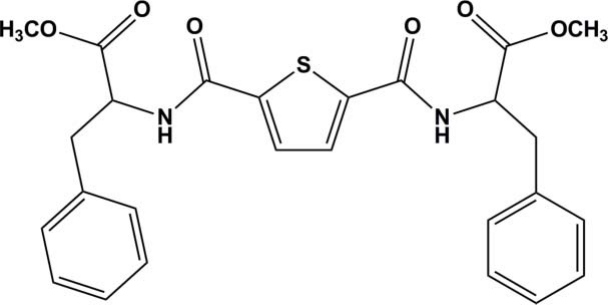


## Experimental

### 

#### Crystal data

C_26_H_26_N_2_O_6_S
*M* *_r_* = 494.56
Orthorhombic, 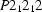

*a* = 9.0769 (3) Å
*b* = 29.6371 (7) Å
*c* = 9.3767 (2) Å
*V* = 2522.45 (12) Å^3^
*Z* = 4
Mo *K*α radiation
μ = 0.17 mm^−1^
*T* = 120 K
0.36 × 0.24 × 0.10 mm

#### Data collection

Oxford Diffraction Xcalibur Eos Gemini diffractometer
Absorption correction: multi-scan (*CrysAlis PRO* RED; Oxford Diffraction, 2009 ▶) *T*_min_ = 0.867, *T*_max_ = 1.000
6802 measured reflections
4233 independent reflections
3315 reflections with *I* > 2σ(*I*)
*R*_int_ = 0.051

#### Refinement

*R*[*F* ^2^ > 2σ(*F* ^2^)] = 0.068
*wR*(*F* ^2^) = 0.187
*S* = 1.10
4233 reflections
288 parameters
1 restraint
H-atom parameters constrained
Δρ_max_ = 0.90 e Å^−3^
Δρ_min_ = −0.70 e Å^−3^
Absolute structure: Flack (1983 ▶), 1669 Friedel pairs
Flack parameter: 0.00 (18)

### 

Data collection: *CrysAlis PRO CCD* (Oxford Diffraction, 2009 ▶); cell refinement: *CrysAlis PRO CCD*; data reduction: *CrysAlis PRO RED* (Oxford Diffraction, 2009 ▶); program(s) used to solve structure: *SHELXS97* (Sheldrick, 2008 ▶); program(s) used to refine structure: *SHELXL97* (Sheldrick, 2008 ▶); molecular graphics: *ORTEP-3 for Windows* (Farrugia, 1997 ▶); software used to prepare material for publication: *SHELXL97*.

## Supplementary Material

Crystal structure: contains datablocks global, I. DOI: 10.1107/S1600536810033210/xu5003sup1.cif

Structure factors: contains datablocks I. DOI: 10.1107/S1600536810033210/xu5003Isup2.hkl

Additional supplementary materials:  crystallographic information; 3D view; checkCIF report

## Figures and Tables

**Table 1:** Hydrogen-bond geometry (Å, °)

*D*—H⋯*A*	*D*—H	H⋯*A*	*D*⋯*A*	*D*—H⋯*A*
N1—H1⋯O6	0.86	2.01	2.853 (5)	164
N2—H2⋯O3^i^	0.86	2.10	2.803 (5)	139

Symmetry code: (i) 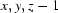.

**Fig. 1.**
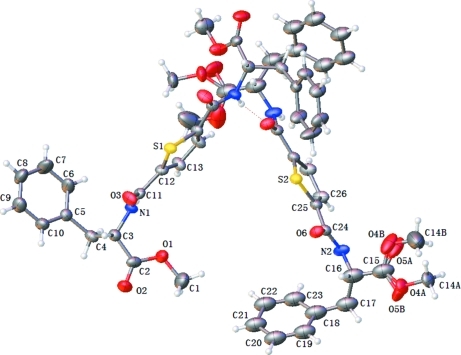
Molecular structure with thermal ellipsoids at 30% probability levels.

**Fig. 2.**
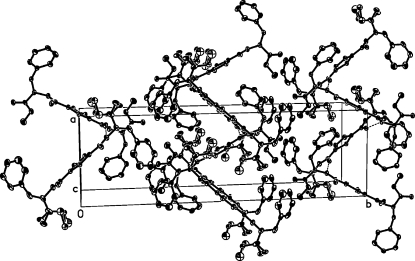
A packing diagram of the title compound along c axis.

## supplementary crystallographic information

### Comment

The thiophene derivates have been viewed as significant compounds for application in many fields (Xia *et al.*, 2010). The title compound derives from natural amino acids. This makes this kind of compounds promising for biological activities.

In the structure of the title compound, the carboxamide groups are approximately coplanar with thiophene ring, and the dihedral angle between thiophene ring and carboxamide is 3.2 (6)°. Title molecules are connected by intermolecular N—H···O hydrogen-bonding interactions forming a supramolecular frameworks. C3 and C16 are chiral atoms in the structure. And the chiral C atom which derived from *L*-phenylalanine kept its known S configuration for that the synthesis reaction did not befallen on the chiral C atom.

### Experimental

2,5-Thiophenedicarboxylic acid (0.3 mmol), thionyl chloride (3 mmol) and 3–5 drops *N*,*N*-dimethylformamide in a flask was heated to 343 K for 10 h. The resulting solution was evaporated under vacuum, and then pale yellow solution of 2,5-thiophenedicarbonyldichloride was obtained.

To a stirred mixture of *L*-phenylalanine methyl ester hydrochloride (129.4 mg, 0.6 mmol) in 15 ml of dry dichloromethane and triethylamine (0.21 ml, 1.5 mmol), 2,5-thiophenedicarbonyldichloride (62.7 mg, 0.3 mmol) in dichloromethane (3 ml) was added dropwise at 253 K and then 20 h at 293 K. The resulting mixture was diluted with dichloromethane, washed with saturated NaHCO_3_ solution and brine, and then dried over anhydrous MgSO_4_. The solvent was condensed *in vacuo*. The title compound was isolated as a white solid by crystallization from 2-propanol (yield: 129.6 mg, 78%). Then the product was recrystallized from THF.

### Refinement

All H atoms were placed in idealized positions and refined using a riding model, with N–H = 0.86 Å, C–H = 0.93–0.98 Å and with *U*_iso_(H) = 1.2–1.5 *U*_eq_(C,N).

### Crystal data

**Table:**

C_26_H_26_N_2_O_6_S	*F*(000) = 1040
*M**_r_* = 494.56	*D*_x_ = 1.302 Mg m^−^^3^
Orthorhombic, *P*2_1_2_1_2	Mo *K*α radiation, λ = 0.71073 Å
Hall symbol: P 2 2ab	Cell parameters from 4047 reflections
*a* = 9.0769 (3) Å	θ = 3.4–25.3°
*b* = 29.6371 (7) Å	µ = 0.17 mm^−^^1^
*c* = 9.3767 (2) Å	*T* = 120 K
*V* = 2522.45 (12) Å^3^	Block, colourless
*Z* = 4	0.36 × 0.24 × 0.10 mm

### Data collection

**Table:**

Oxford Diffraction Xcalibur Eos Gemini diffractometer	4233 independent reflections
Radiation source: Enhance (Mo) X-ray Source	3315 reflections with *I* > 2σ(*I*)
graphite	*R*_int_ = 0.051
Detector resolution: 16.0355 pixels mm^-1^	θ_max_ = 25.0°, θ_min_ = 3.4°
ω scans	*h* = −6→10
Absorption correction: multi-scan (*CrysAlis PRO* RED; Oxford Diffraction, 2009)	*k* = −28→35
*T*_min_ = 0.867, *T*_max_ = 1.000	*l* = −7→11
6802 measured reflections	

### Refinement

**Table:**

Refinement on *F*^2^	Secondary atom site location: difference Fourier map
Least-squares matrix: full	Hydrogen site location: inferred from neighbouring sites
*R*[*F*^2^ > 2σ(*F*^2^)] = 0.068	H-atom parameters constrained
*wR*(*F*^2^) = 0.187	*w* = 1/[σ^2^(*F*_o_^2^) + (0.1124*P*)^2^ + 0.8367*P*] where *P* = (*F*_o_^2^ + 2*F*_c_^2^)/3
*S* = 1.10	(Δ/σ)_max_ < 0.001
4233 reflections	Δρ_max_ = 0.90 e Å^−^^3^
288 parameters	Δρ_min_ = −0.70 e Å^−^^3^
1 restraint	Absolute structure: Flack (1983), 1669 Friedel pairs
Primary atom site location: structure-invariant direct methods	Flack parameter: 0.00 (18)

### Special details

**Table:**

Geometry. All e.s.d.'s (except the e.s.d. in the dihedral angle between two l.s. planes) are estimated using the full covariance matrix. The cell e.s.d.'s are taken into account individually in the estimation of e.s.d.'s in distances, angles and torsion angles; correlations between e.s.d.'s in cell parameters are only used when they are defined by crystal symmetry. An approximate (isotropic) treatment of cell e.s.d.'s is used for estimating e.s.d.'s involving l.s. planes.
Refinement. Refinement of *F*^2^ against ALL reflections. The weighted *R*-factor *wR* and goodness of fit *S* are based on *F*^2^, conventional *R*-factors *R* are based on *F*, with *F* set to zero for negative *F*^2^. The threshold expression of *F*^2^ > σ(*F*^2^) is used only for calculating *R*-factors(gt) *etc*. and is not relevant to the choice of reflections for refinement. *R*-factors based on *F*^2^ are statistically about twice as large as those based on *F*, and *R*- factors based on ALL data will be even larger.

### Fractional atomic coordinates and isotropic or equivalent isotropic displacement parameters (Å^2^)

**Table:**

	*x*	*y*	*z*	*U*_iso_*/*U*_eq_	Occ. (<1)
C1	0.3969 (8)	0.6948 (2)	0.3748 (8)	0.0676 (18)	
H1A	0.3112	0.6771	0.3529	0.101*	
H1B	0.4259	0.7117	0.2920	0.101*	
H1C	0.3745	0.7153	0.4512	0.101*	
C2	0.6263 (6)	0.68418 (15)	0.4908 (5)	0.0361 (11)	
C3	0.7416 (5)	0.65073 (14)	0.5404 (5)	0.0336 (11)	
H3	0.7545	0.6551	0.6432	0.040*	
C4	0.8884 (6)	0.66076 (14)	0.4700 (5)	0.0338 (11)	
H4B	0.9036	0.6932	0.4685	0.041*	
H4A	0.8855	0.6503	0.3720	0.041*	
C5	1.0183 (6)	0.63839 (14)	0.5462 (4)	0.0325 (10)	
C6	1.0574 (6)	0.59423 (18)	0.5155 (7)	0.0529 (15)	
H6	1.0076	0.5782	0.4451	0.063*	
C7	1.1729 (8)	0.5739 (2)	0.5915 (8)	0.069 (2)	
H7	1.2017	0.5446	0.5691	0.083*	
C8	1.2438 (6)	0.59686 (19)	0.6990 (7)	0.0549 (15)	
H8	1.3177	0.5828	0.7514	0.066*	
C9	1.2052 (7)	0.64021 (18)	0.7282 (6)	0.0521 (14)	
H9	1.2551	0.6562	0.7985	0.063*	
C10	1.0909 (6)	0.66065 (16)	0.6527 (5)	0.0433 (13)	
H10	1.0635	0.6900	0.6753	0.052*	
C11	0.6350 (5)	0.57915 (14)	0.6197 (4)	0.0281 (10)	
C12	0.5668 (5)	0.53566 (13)	0.5707 (4)	0.0281 (10)	
C13	0.5371 (6)	0.52036 (15)	0.4374 (4)	0.0352 (12)	
H13	0.5639	0.5357	0.3548	0.042*	
C14A	0.207 (2)	0.6027 (7)	0.130 (2)	0.047 (4)	0.275 (12)
H14B	0.1740	0.5900	0.0411	0.070*	0.275 (12)
H14C	0.1380	0.6250	0.1616	0.070*	0.275 (12)
H14A	0.2147	0.5792	0.1999	0.070*	0.275 (12)
C14B	0.3361 (17)	0.5483 (4)	0.091 (2)	0.123 (6)	0.725 (12)
H14E	0.2938	0.5485	−0.0026	0.184*	0.725 (12)
H14F	0.2793	0.5674	0.1529	0.184*	0.725 (12)
H14D	0.3359	0.5181	0.1280	0.184*	0.725 (12)
C15	0.4767 (9)	0.6062 (2)	0.0647 (6)	0.0674 (7)	
C16	0.6251 (9)	0.6243 (2)	0.0738 (7)	0.0674 (7)	
H16	0.6438	0.6299	0.1752	0.081*	
C17	0.6498 (9)	0.6692 (2)	−0.0017 (7)	0.0674 (7)	
H17A	0.6582	0.6638	−0.1034	0.081*	
H17B	0.5646	0.6883	0.0137	0.081*	
C18	0.7826 (9)	0.6931 (2)	0.0477 (6)	0.0674 (7)	
C19	0.7774 (9)	0.7261 (2)	0.1503 (6)	0.0674 (7)	
H19	0.6853	0.7354	0.1822	0.081*	
C20	0.9003 (9)	0.7463 (2)	0.2091 (7)	0.0674 (7)	
H20	0.8902	0.7686	0.2783	0.081*	
C21	1.0373 (9)	0.7331 (2)	0.1643 (6)	0.0674 (7)	
H21	1.1207	0.7472	0.2008	0.081*	
C22	1.0519 (9)	0.6990 (2)	0.0649 (6)	0.0674 (7)	
H22	1.1448	0.6886	0.0389	0.081*	
C23	0.9280 (9)	0.6808 (2)	0.0052 (7)	0.0674 (7)	
H23	0.9391	0.6594	−0.0666	0.081*	
C24	0.8109 (6)	0.56624 (14)	0.1212 (4)	0.0287 (10)	
C25	0.9113 (5)	0.53145 (14)	0.0658 (4)	0.0284 (10)	
C26	0.9493 (6)	0.51816 (16)	−0.0702 (4)	0.0395 (13)	
H26	0.9124	0.5315	−0.1526	0.047*	
N1	0.6976 (4)	0.60424 (11)	0.5186 (4)	0.0276 (8)	
H1	0.7127	0.5923	0.4364	0.033*	
N2	0.7346 (5)	0.59066 (12)	0.0283 (4)	0.0378 (10)	
H2	0.7497	0.5867	−0.0614	0.045*	
O1	0.5160 (5)	0.66529 (11)	0.4175 (4)	0.0507 (10)	
O2	0.6327 (4)	0.72365 (11)	0.5165 (5)	0.0555 (11)	
O3	0.6321 (4)	0.59001 (10)	0.7462 (3)	0.0403 (9)	
O4A	0.342 (2)	0.6223 (6)	0.111 (2)	0.047 (4)	0.275 (12)
O4B	0.4741 (16)	0.5635 (4)	0.0848 (17)	0.078 (3)	0.725 (12)
O5A	0.443 (6)	0.5668 (8)	0.057 (5)	0.078 (3)	0.275 (12)
O5B	0.3754 (8)	0.6338 (3)	0.0463 (11)	0.070 (2)	0.725 (12)
O6	0.8024 (4)	0.57171 (10)	0.2509 (3)	0.0349 (8)	
S1	0.5000	0.5000	0.70005 (14)	0.0268 (4)	
S2	1.0000	0.5000	0.19249 (14)	0.0265 (4)	

### Atomic displacement parameters (Å^2^)

**Table:**

	*U*^11^	*U*^22^	*U*^33^	*U*^12^	*U*^13^	*U*^23^
C1	0.063 (4)	0.068 (4)	0.072 (4)	0.008 (3)	−0.025 (4)	−0.005 (3)
C2	0.042 (3)	0.030 (2)	0.037 (2)	0.007 (2)	0.012 (2)	0.0014 (19)
C3	0.041 (3)	0.033 (2)	0.027 (2)	−0.002 (2)	−0.002 (2)	−0.0013 (18)
C4	0.050 (3)	0.029 (2)	0.022 (2)	−0.007 (2)	0.000 (2)	−0.0004 (17)
C5	0.033 (3)	0.034 (2)	0.030 (2)	−0.001 (2)	0.004 (2)	−0.0016 (18)
C6	0.048 (3)	0.051 (3)	0.059 (3)	0.006 (3)	−0.013 (3)	−0.030 (3)
C7	0.073 (4)	0.055 (3)	0.081 (4)	0.031 (3)	−0.026 (4)	−0.038 (3)
C8	0.046 (3)	0.059 (3)	0.060 (3)	0.018 (3)	−0.018 (3)	−0.013 (3)
C9	0.054 (4)	0.052 (3)	0.051 (3)	0.004 (3)	−0.009 (3)	−0.020 (3)
C10	0.053 (3)	0.033 (2)	0.044 (3)	0.003 (2)	−0.009 (3)	−0.008 (2)
C11	0.035 (3)	0.031 (2)	0.018 (2)	0.004 (2)	−0.0055 (19)	0.0008 (16)
C12	0.041 (3)	0.027 (2)	0.0163 (19)	0.002 (2)	0.0030 (19)	0.0048 (16)
C13	0.056 (4)	0.035 (2)	0.015 (2)	−0.012 (2)	−0.005 (2)	0.0033 (17)
C14A	0.034 (7)	0.053 (8)	0.053 (8)	−0.013 (5)	0.010 (6)	0.002 (6)
C14B	0.110 (12)	0.068 (7)	0.191 (16)	−0.018 (7)	−0.066 (11)	0.014 (8)
C15	0.0996 (18)	0.0573 (12)	0.0455 (11)	0.0261 (13)	0.0108 (12)	−0.0013 (9)
C16	0.0996 (18)	0.0573 (12)	0.0455 (11)	0.0261 (13)	0.0108 (12)	−0.0013 (9)
C17	0.0996 (18)	0.0573 (12)	0.0455 (11)	0.0261 (13)	0.0108 (12)	−0.0013 (9)
C18	0.0996 (18)	0.0573 (12)	0.0455 (11)	0.0261 (13)	0.0108 (12)	−0.0013 (9)
C19	0.0996 (18)	0.0573 (12)	0.0455 (11)	0.0261 (13)	0.0108 (12)	−0.0013 (9)
C20	0.0996 (18)	0.0573 (12)	0.0455 (11)	0.0261 (13)	0.0108 (12)	−0.0013 (9)
C21	0.0996 (18)	0.0573 (12)	0.0455 (11)	0.0261 (13)	0.0108 (12)	−0.0013 (9)
C22	0.0996 (18)	0.0573 (12)	0.0455 (11)	0.0261 (13)	0.0108 (12)	−0.0013 (9)
C23	0.0996 (18)	0.0573 (12)	0.0455 (11)	0.0261 (13)	0.0108 (12)	−0.0013 (9)
C24	0.040 (3)	0.026 (2)	0.020 (2)	−0.002 (2)	0.000 (2)	−0.0021 (17)
C25	0.039 (3)	0.024 (2)	0.022 (2)	−0.0018 (19)	0.000 (2)	0.0001 (16)
C26	0.069 (4)	0.036 (2)	0.013 (2)	0.008 (2)	−0.002 (2)	0.0005 (16)
N1	0.044 (2)	0.0221 (16)	0.0167 (16)	−0.0009 (17)	−0.0006 (17)	−0.0027 (14)
N2	0.065 (3)	0.0338 (19)	0.0141 (16)	0.018 (2)	−0.0050 (19)	−0.0061 (14)
O1	0.054 (2)	0.0389 (18)	0.059 (2)	0.0089 (18)	−0.026 (2)	−0.0115 (16)
O2	0.057 (3)	0.0316 (19)	0.078 (3)	0.0055 (18)	−0.012 (2)	−0.0154 (17)
O3	0.066 (2)	0.0395 (17)	0.0156 (15)	−0.0111 (17)	−0.0077 (16)	−0.0038 (13)
O4A	0.034 (7)	0.053 (8)	0.053 (8)	−0.013 (5)	0.010 (6)	0.002 (6)
O4B	0.047 (8)	0.115 (4)	0.072 (8)	0.011 (4)	−0.005 (4)	0.048 (4)
O5A	0.047 (8)	0.115 (4)	0.072 (8)	0.011 (4)	−0.005 (4)	0.048 (4)
O5B	0.041 (4)	0.070 (5)	0.100 (6)	0.020 (4)	−0.026 (4)	−0.014 (4)
O6	0.049 (2)	0.0383 (16)	0.0170 (15)	0.0106 (16)	−0.0037 (14)	−0.0017 (12)
S1	0.0425 (9)	0.0263 (7)	0.0118 (6)	0.0010 (7)	0.000	0.000
S2	0.0384 (8)	0.0268 (7)	0.0144 (6)	0.0011 (7)	0.000	0.000

### Geometric parameters (Å, °)

**Table:**

C1—O1	1.447 (7)	C14B—H14E	0.9600
C1—H1A	0.9600	C14B—H14F	0.9600
C1—H1B	0.9600	C14B—H14D	0.9600
C1—H1C	0.9600	C15—O5A	1.21 (2)
C2—O2	1.196 (5)	C15—O5B	1.243 (9)
C2—O1	1.337 (6)	C15—O4B	1.280 (12)
C2—C3	1.515 (7)	C15—O4A	1.38 (2)
C3—N1	1.449 (6)	C15—C16	1.453 (11)
C3—C4	1.516 (7)	C16—N2	1.471 (7)
C3—H3	0.9800	C16—C17	1.523 (9)
C4—C5	1.530 (7)	C16—H16	0.9800
C4—H4B	0.9700	C17—C18	1.472 (10)
C4—H4A	0.9700	C17—H17A	0.9700
C5—C10	1.366 (7)	C17—H17B	0.9700
C5—C6	1.386 (7)	C18—C19	1.373 (8)
C6—C7	1.404 (9)	C18—C23	1.426 (10)
C6—H6	0.9300	C19—C20	1.381 (10)
C7—C8	1.376 (8)	C19—H19	0.9300
C7—H7	0.9300	C20—C21	1.369 (10)
C8—C9	1.360 (8)	C20—H20	0.9300
C8—H8	0.9300	C21—C22	1.381 (9)
C9—C10	1.395 (8)	C21—H21	0.9300
C9—H9	0.9300	C22—C23	1.367 (10)
C10—H10	0.9300	C22—H22	0.9300
C11—O3	1.229 (5)	C23—H23	0.9300
C11—N1	1.332 (6)	C24—O6	1.229 (5)
C11—C12	1.502 (6)	C24—N2	1.328 (6)
C12—C13	1.357 (6)	C24—C25	1.471 (6)
C12—S1	1.719 (4)	C25—C26	1.378 (6)
C13—C13^i^	1.383 (9)	C25—S2	1.711 (4)
C13—H13	0.9300	C26—C26^ii^	1.417 (10)
C14A—O4A	1.37 (3)	C26—H26	0.9300
C14A—H14B	0.9600	N1—H1	0.8600
C14A—H14C	0.9600	N2—H2	0.8600
C14A—H14A	0.9600	S1—C12^i^	1.719 (4)
C14B—O4B	1.33 (2)	S2—C25^ii^	1.711 (4)
			
O1—C1—H1A	109.5	O5A—C15—O4A	97 (3)
O1—C1—H1B	109.5	O4B—C15—O4A	106.1 (13)
H1A—C1—H1B	109.5	O5A—C15—C16	126 (3)
O1—C1—H1C	109.5	O5B—C15—C16	116.8 (7)
H1A—C1—H1C	109.5	O4B—C15—C16	112.0 (8)
H1B—C1—H1C	109.5	O4A—C15—C16	132.5 (9)
O2—C2—O1	123.4 (5)	C15—C16—N2	111.0 (5)
O2—C2—C3	123.0 (5)	C15—C16—C17	115.6 (6)
O1—C2—C3	113.6 (4)	N2—C16—C17	110.9 (6)
N1—C3—C2	112.9 (4)	C15—C16—H16	106.2
N1—C3—C4	111.6 (4)	N2—C16—H16	106.2
C2—C3—C4	110.2 (4)	C17—C16—H16	106.2
N1—C3—H3	107.3	C18—C17—C16	113.2 (6)
C2—C3—H3	107.3	C18—C17—H17A	108.9
C4—C3—H3	107.3	C16—C17—H17A	108.9
C3—C4—C5	112.9 (4)	C18—C17—H17B	108.9
C3—C4—H4B	109.0	C16—C17—H17B	108.9
C5—C4—H4B	109.0	H17A—C17—H17B	107.7
C3—C4—H4A	109.0	C19—C18—C23	114.1 (7)
C5—C4—H4A	109.0	C19—C18—C17	122.4 (7)
H4B—C4—H4A	107.8	C23—C18—C17	123.2 (5)
C10—C5—C6	119.0 (5)	C18—C19—C20	124.2 (7)
C10—C5—C4	120.3 (4)	C18—C19—H19	117.9
C6—C5—C4	120.6 (4)	C20—C19—H19	117.9
C5—C6—C7	119.4 (5)	C21—C20—C19	119.1 (6)
C5—C6—H6	120.3	C21—C20—H20	120.4
C7—C6—H6	120.3	C19—C20—H20	120.4
C8—C7—C6	120.5 (5)	C20—C21—C22	120.2 (7)
C8—C7—H7	119.7	C20—C21—H21	119.9
C6—C7—H7	119.7	C22—C21—H21	119.9
C9—C8—C7	119.7 (5)	C23—C22—C21	119.0 (7)
C9—C8—H8	120.2	C23—C22—H22	120.5
C7—C8—H8	120.2	C21—C22—H22	120.5
C8—C9—C10	120.0 (5)	C22—C23—C18	123.2 (6)
C8—C9—H9	120.0	C22—C23—H23	118.4
C10—C9—H9	120.0	C18—C23—H23	118.4
C5—C10—C9	121.3 (4)	O6—C24—N2	123.0 (4)
C5—C10—H10	119.3	O6—C24—C25	118.7 (4)
C9—C10—H10	119.3	N2—C24—C25	118.3 (4)
O3—C11—N1	123.3 (4)	C26—C25—C24	133.0 (4)
O3—C11—C12	120.7 (4)	C26—C25—S2	111.7 (3)
N1—C11—C12	115.9 (4)	C24—C25—S2	115.4 (3)
C13—C12—C11	130.6 (4)	C25—C26—C26^ii^	112.3 (3)
C13—C12—S1	112.0 (3)	C25—C26—H26	123.8
C11—C12—S1	117.2 (3)	C26^ii^—C26—H26	123.8
C12—C13—C13^i^	112.9 (3)	C11—N1—C3	123.2 (4)
C12—C13—H13	123.6	C11—N1—H1	118.4
C13^i^—C13—H13	123.6	C3—N1—H1	118.4
O4A—C14A—H14B	109.5	C24—N2—C16	122.1 (4)
O4A—C14A—H14C	109.5	C24—N2—H2	119.0
H14B—C14A—H14C	109.5	C16—N2—H2	119.0
O4A—C14A—H14A	109.5	C2—O1—C1	116.6 (4)
H14B—C14A—H14A	109.5	C14A—O4A—C15	133.5 (17)
H14C—C14A—H14A	109.5	C15—O4B—C14B	111.0 (12)
O5A—C15—O5B	116 (2)	C12^i^—S1—C12	90.3 (3)
O5B—C15—O4B	131.1 (11)	C25^ii^—S2—C25	92.1 (3)
			
O2—C2—C3—N1	169.7 (4)	C17—C18—C19—C20	173.7 (6)
O1—C2—C3—N1	−9.4 (6)	C18—C19—C20—C21	−0.1 (10)
O2—C2—C3—C4	−64.8 (6)	C19—C20—C21—C22	−2.1 (9)
O1—C2—C3—C4	116.1 (4)	C20—C21—C22—C23	4.4 (9)
N1—C3—C4—C5	−71.2 (4)	C21—C22—C23—C18	−4.6 (10)
C2—C3—C4—C5	162.7 (4)	C19—C18—C23—C22	2.4 (9)
C3—C4—C5—C10	−90.3 (5)	C17—C18—C23—C22	−171.2 (6)
C3—C4—C5—C6	85.1 (6)	O6—C24—C25—C26	−177.5 (5)
C10—C5—C6—C7	−1.7 (9)	N2—C24—C25—C26	1.8 (8)
C4—C5—C6—C7	−177.2 (6)	O6—C24—C25—S2	3.2 (6)
C5—C6—C7—C8	2.2 (11)	N2—C24—C25—S2	−177.5 (4)
C6—C7—C8—C9	−2.4 (11)	C24—C25—C26—C26^ii^	−179.0 (5)
C7—C8—C9—C10	2.2 (10)	S2—C25—C26—C26^ii^	0.4 (8)
C6—C5—C10—C9	1.5 (8)	O3—C11—N1—C3	−13.2 (7)
C4—C5—C10—C9	177.0 (5)	C12—C11—N1—C3	167.1 (4)
C8—C9—C10—C5	−1.8 (9)	C2—C3—N1—C11	−95.6 (5)
O3—C11—C12—C13	168.5 (5)	C4—C3—N1—C11	139.6 (4)
N1—C11—C12—C13	−11.8 (8)	O6—C24—N2—C16	−4.5 (8)
O3—C11—C12—S1	−5.5 (6)	C25—C24—N2—C16	176.3 (5)
N1—C11—C12—S1	174.2 (3)	C15—C16—N2—C24	−99.5 (6)
C11—C12—C13—C13^i^	−175.3 (5)	C17—C16—N2—C24	130.5 (6)
S1—C12—C13—C13^i^	−1.0 (8)	O2—C2—O1—C1	−3.0 (8)
O5A—C15—C16—N2	18 (3)	C3—C2—O1—C1	176.1 (5)
O5B—C15—C16—N2	−153.2 (7)	O5A—C15—O4A—C14A	−6(3)
O4B—C15—C16—N2	30.1 (11)	O5B—C15—O4A—C14A	122 (3)
O4A—C15—C16—N2	171.0 (12)	O4B—C15—O4A—C14A	−22 (3)
O5A—C15—C16—C17	145 (3)	C16—C15—O4A—C14A	−164.4 (18)
O5B—C15—C16—C17	−25.7 (9)	O5A—C15—O4B—C14B	−41 (11)
O4B—C15—C16—C17	157.6 (10)	O5B—C15—O4B—C14B	−3(2)
O4A—C15—C16—C17	−61.5 (14)	O4A—C15—O4B—C14B	22.3 (18)
C15—C16—C17—C18	161.2 (5)	C16—C15—O4B—C14B	173.4 (12)
N2—C16—C17—C18	−71.2 (7)	C13—C12—S1—C12^i^	0.4 (3)
C16—C17—C18—C19	−96.0 (7)	C11—C12—S1—C12^i^	175.5 (5)
C16—C17—C18—C23	77.1 (8)	C26—C25—S2—C25^ii^	−0.1 (3)
C23—C18—C19—C20	0.0 (9)	C24—C25—S2—C25^ii^	179.3 (5)

Symmetry codes: (i) −*x*+1, −*y*+1, *z*; (ii) −*x*+2, −*y*+1, *z*.

### Hydrogen-bond geometry (Å, °)

**Table:**

*D*—H···*A*	*D*—H	H···*A*	*D*···*A*	*D*—H···*A*
N1—H1···O6	0.86	2.01	2.853 (5)	164.
N2—H2···O3^iii^	0.86	2.10	2.803 (5)	139.

Symmetry codes: (iii) *x*, *y*, *z*−1.
